# Secondhand Smoke Exposure and Smoking Prevalence Among Adolescents

**DOI:** 10.1001/jamanetworkopen.2023.38166

**Published:** 2023-10-20

**Authors:** Yuki Kuwabara, Aya Kinjo, Hongja Kim, Ruriko Minobe, Hitoshi Maesato, Susumu Higuchi, Hisashi Yoshimoto, Maki Jike, Yuichiro Otsuka, Osamu Itani, Yoshitaka Kaneita, Hideyuki Kanda, Hideaki Kasuga, Teruna Ito, Yoneatsu Osaki

**Affiliations:** 1 Division of Environmental and Preventive Medicine, Department of Social Medicine, Faculty of Medicine, Tottori University, Tottori, Japan; 2 National Institute of Alcoholism, Kurihama National Hospital, Kanagawa, Japan; 3 Primary Care and Medical Education, Graduate School of Comprehensive Human Sciences, Majors of Medical Science, University of Tsukuba, Ibaragi, Japan; 4 Department of Food Science and Nutrition, Faculty of Life and Environmental Science, Showa Women’s University, Tokyo, Japan; 5 Department of Public Health, School of Medicine, Nihon University, Tokyo, Japan; 6 Dentistry and Pharmaceutical Sciences, Department of Public Health, Okayama University Graduate School of Medicine, Okayama, Japan; 7 Department of Hygiene and Preventive Medicine, Fukushima Medical University, Fukushima, Japan; 8 Department of Food and Nutrition, Koriyama Women’s University, Koriyama, Japan

## Abstract

**Importance:**

Secondhand smoke is a substantial risk factor for youth health globally, including in Japan, where tobacco control policies should be reassessed.

**Objective:**

To assess trends in the prevalence of secondhand smoke exposure among Japanese adolescents from 2008 to 2017 and to examine the association between its frequency and smoking during the study period.

**Design, Setting, and Participants:**

This study comprised a nationally representative, self-administered, school-based, cross-sectional survey focusing on tobacco and alcohol use and related factors among students in grades 7 to 12 (ages 12-18 years) in Japan. This random sampling survey used single-stage cluster sampling. Using the national school directory, junior and senior high schools throughout Japan were randomly extracted from each regional block. All students enrolled in the sampled schools were included as participants, and school-based surveys were completed in 2008, 2012, and 2017. Data analysis was performed from January 1 to March 15, 2023.

**Main Outcomes and Measures:**

Prevalence of secondhand smoke exposure among adolescents in Japan from 2008 to 2017 and changes in the association between secondhand smoke exposure frequency and prevalence of smoking were examined using multiple logistic regression analysis.

**Results:**

Data were analyzed for 95 680 adolescents in 2008 (50.7% boys), 100 050 in 2012 (51.6% boys), and 64 152 in 2017 (53.9% boys). At baseline, 42.0%, 38.5%, and 34.6% of the participants were junior high school students in 2008, 2012, and 2017, respectively. Based on the 2008 surveys, 51.0% of adolescents in grades 7 to 12 were exposed to secondhand smoke in any place (≥1 day during the past 7 days), 37.2% were exposed at home, and 36.5% were exposed in public places. In 2017, 36.3% of participants were exposed to secondhand smoke in any place, 23.8% were exposed at home, and 27.0% were exposed in public places. An association between secondhand smoke exposure frequency and prevalence of smoking was observed consistently regardless of survey year, location, or pattern of exposure (adjusted odds ratios ranged from 2.29 [95% CI, 1.81-2.91] for 1-2 days at home to 11.15 [95% CI, 8.50-14.62] for 7 days in public places). Stratified analysis by higher education intention indicated that the prevalence of smoking and secondhand smoke exposure decreased but remained higher among adolescents who did not intend to pursue higher education. The association between secondhand smoke and smoking did not differ substantially between groups.

**Conclusions and Relevance:**

In this cross-sectional study, the prevalence of secondhand smoke exposure among adolescents in Japan decreased but remained at high levels overall. There may not be a hazard-free threshold for smoking. Enhancing comprehensive tobacco control strategies is Japan’s first step toward achieving smoke-free environments to protect youths. Implementation and verification of the effectiveness of smoke-free legislation should be considered.

## Key Points

**Question:**

What percentage of Japanese adolescents were exposed to secondhand smoke from 2008 to 2017, and is there an association between that exposure and smoking?

**Findings:**

In this cross-sectional study of 95 680, 100 050, and 64 152 Japanese adolescents surveyed in 2008, 2012, and 2017, respectively, secondhand smoke exposure decreased, but approximately one-third of participants were still exposed to smoke in 2017. The association between secondhand smoke exposure frequency and smoking was consistently statistically significant regardless of survey year, location, or socioeconomic group.

**Meaning:**

To protect youths from secondhand smoke, Japan should enhance comprehensive tobacco control strategies to match the global standard and consider legislation to achieve a smoke-free environment.

## Teaser

This cross-sectional study assesses trends in secondhand smoke exposure prevalence among Japanese adolescents between 2008 and 2017 and subsequent associations between secondhand smoke exposure frequency and smoking status.

## Introduction

Secondhand smoke (SHS) exposure is a key modifiable risk factor for youth health burden.^1^ Reducing unwanted passive smoking could substantially contribute to health gains. In 2004, the World Health Organization estimated that SHS caused 165 000 deaths through lower respiratory infections in children aged younger than 5 years and 1150 asthma-related deaths in those aged younger than 15 years. The links between SHS and specific diseases have been summarized in comprehensive assessments and reviews.^2,3,4,5,6^ In children, exposure to SHS is associated with an increased prevalence of respiratory infection, increased frequency and severity of asthma exacerbations, and greater risk of sudden infant death syndrome.^7^ The existing literature suggests that SHS exposure among children and youths could affect their cardiovascular health and cancer mortality.^8,9^ The American Heart Association strongly warns against SHS exposure and calls for stricter smoke-free legislation to prevent SHS exposure among children and youths.

Despite global efforts to develop comprehensive tobacco control strategies, such as the Framework Convention on Tobacco Control (FCTC), a considerable number of individuals remain unprotected from SHS exposure.^10^ The 2010 to 2018 Global Youth Tobacco Survey surveyed adolescents aged between 12 and 16 years in 142 countries, and researchers found that 57.6% and 33.1% were exposed to SHS for at least 1 day during the past 7 days in public places and at home, respectively.^11^ The US Centers for Disease Control and Prevention (CDC) reported that although SHS exposure decreased from 1988 to 2014, 25% of nonsmokers, including 14 million children, were exposed to SHS.^12^ According to the 2013 to 2016 National Health and Nutrition Examination Survey, 35.4% of nonsmokers aged 3 to 17 years in the US are exposed to SHS.^13^ Measures against SHS remain a key challenge both for countries with advanced tobacco control policies and for those lacking such policies. Although Japan’s insufficient tobacco control became an issue during the 2020 Tokyo Olympics,^14^ no reports have yet examined the prevalence of SHS exposure among Japanese adolescents based on nationally representative surveys.

Secondhand smoke exposure in adolescence may influence smoking behavior.^15^ Studies indicate that SHS in childhood is an independent factor for susceptibility to smoking initiation.^16^ Children who live with smoking families are more likely to be exposed to SHS and smoke tobacco later in life compared with children who live with nonsmoking families.^17^ However, to our knowledge, no studies have examined how SHS exposure frequency and location are associated with adolescent smoking behavior.

Regarding socioeconomic factors, a review reported that parental smoking, lower socioeconomic status (SES), and lower educational attainment are consistently associated with SHS exposure among children and youths.^18^ The association between SHS exposure and SES is supported by studies from many countries, including countries in Asia.^19,20,21,22,23,24^ However, studies of socioeconomic disparities in SHS exposure among Japanese adolescents are scarce, except for a study from a rural area.^25^ Examining disparities in the association between SHS exposure and smoking across SES could be relevant to understanding whether social disparities in smoking can be attributed to SHS exposure.

We used data collected in 2008, 2012, and 2017 nationwide surveys of junior high and high school students to assess trends in the prevalence of SHS exposure among Japanese adolescents. We also examined both the association between SHS exposure frequency and smoking over the study period and disparities in the association between SHS exposure and smoking across social backgrounds by using further education intention as a marker for household SES over time.

## Methods

### Study Design and Participants

This cross-sectional study incorporated a nationally representative, self-administered, school-based survey mainly focusing on tobacco and alcohol use and related factors among students in grades 7 to 12 (ages 12-18 years) in Japan. This random sampling survey used single-stage cluster sampling.^26^ Using the national school directory, junior and senior high schools throughout Japan were randomly extracted from each regional block. All students enrolled in the sampled schools were included as participants. After we obtained agreement to participate from school principals, we sent survey questionnaires to all students at the school. Schools provided parents or caregivers with information about our survey and the opportunity to withdraw from this study. Then the anonymized questionnaires and envelopes for individual privacy were distributed by classroom teachers. Teachers encouraged students to respond voluntarily and honestly. The completed questionnaires were placed in envelopes and sealed by students; envelopes were collected by teachers and returned to our office. The data collection flow was reported previously.^27^

The Ethical Review Committee of the Faculty of Medicine at Tottori University approved this study and waived the requirement for informed consent because only deidentified data were used. Details are presented in the eMethods in Supplement 1. This study adhered to the Strengthening the Reporting of Observational Studies in Epidemiology (STROBE) reporting guideline.

### Measurements of SHS Exposure and Current Smoking

Students were defined as exposed to SHS at home if they responded “1 or more days” to the question, “During the past 7 days, on how many days have people smoked in your presence, in your home?” Students were defined as exposed to SHS in public places if they who responded “1 or more days” to the question, “During the past 7 days, on how many days have people smoked in your presence, in places other than in your home?” Based on these responses, exposure to SHS in any place was further defined as exposure to SHS in public places or at home on at least 1 day during the past 7 days. The frequency of SHS exposure at home, in public places, or in any place was defined as 1 or more days, 3 or more days, 5 or more days, and daily during the past 7 days, based on student responses. Current smokers were defined as those who responded “at least once” to the question, “How many days in the past 30 days have you smoked cigarettes?”

### Covariates and Further Education Intention

To examine the association between SHS exposure and current smoking status, several variables were categorized into binary or ordinal data and used to adjust for potential confounding factors. The following were examined: demographic variables (sex and grade [7-12]), health behavior variables (having breakfast every day, participating in club activities, and enjoying school), attitude toward tobacco (understanding that secondhand smoking is harmful and understanding that smoking is harmful), and current alcohol use (drinking once in the past 30 days). We used further education intention as a marker for household SES, based on a previous study.^25^ We categorized further education intention into 2 levels (college or higher, or other options), according to the following responses to the question, “What is your intention for after graduating from school?”: “senior high school,” “vocational school,” “junior college,” “college,” “postgraduate school,” “starting a job after graduating current school,” and “undecided.” After discussions within the research team, we assumed that these covariates could be associated with both SHS exposure and smoking, according to the previous literature.^28,29,30,31,32^ The eAppendix in Supplement 1 presents the survey questionnaire and data categorization.

### Statistical Analysis

Prevalence estimates of SHS exposure and 95% CIs were calculated using a weighting method based on single-stage stratified cluster random sampling.^26^ Prevalence was adjusted for grade and sex using the number of junior and senior high school students nationwide, drawn from the School Basic Survey conducted by the Ministry of Education, Culture, Sports, Science and Technology of Japan as a standard population. Participants whose school grades were missing were excluded from the analysis because we determined that the missing data (0.1%-0.3%) did not significantly affect the results. A χ^2^ test was used to test the difference in proportions according to sex and age. Multiple logistic regression analyses were conducted to examine the association between the frequency of SHS exposure (0, 1-2, 3-4, 5-6, or 7 days) and current smoking status. We adjusted for the selected covariates noted earlier. The covariates did not interfere with model fitting or multicollinearity. We set the group with “0 days during the past 7 days” as a reference and calculated the adjusted odds ratios (AORs) and *P* values. Stratified analysis was also conducted according to 2 educational intention levels (college or higher, or other options) to examine the difference in the proportion of current smokers and the AORs for current smoking by SHS exposure frequency levels. Moreover, the association between SHS exposure pattern and current smoking status was examined using logistic regression analysis. The pattern of SHS exposure was categorized into 4 groups according to SHS exposure during the past 7 days at home and in public places (0 days for both, ≥1 day and 0 days, 0 days and ≥1 day, and ≥1 day for both, respectively). We set the group with 0 days at both home and in public places as a reference category in the logistic regression analysis. The significance threshold for statistical tests was set at *P* < .05 (2-sided). Missing data on independent variables were handled by applying multiple imputations with 20 imputations using a fully conditional specification. Data analysis was performed from January 1 to March 15, 2023, using SPSS, version 25.0 (IBM Corp), and Stata, version 16 (StataCorp LLC).

## Results

Details on the surveys, number of schools, school selection and response rates, and participant background are provided in Table 1. Data were analyzed for 95 680 adolescents (50.7% boys and 49.3% girls) in grades 7 to 12 (40 151 junior high school students [42.0%]) in 2008, 100 050 adolescents (51.6% boys and 48.4% girls) in grades 7 to 12 (38 494 junior high school students [38.5%]) in 2012, and 64 152 adolescents (53.9% boys and 46.1% girls) in grades 7 to 12 (22 215 junior high school students [34.6%]) in 2017. The sample included 1.2% and 2.2% of nationwide junior high school and high school students in 2008, 1.4% and 2.7% in 2012, and 0.9% and 1.8% in 2017, respectively.

**Table 1.** School Sampling, Response Rates, and Participant Characteristics by Survey Year^a^

	**2008**	**2012**	**2017**
**Schools in Japan**			
Junior high	10 882	10 018	10 325
Schools sampled	130 (1.2)	140 (1.4)	98 (0.9)
Sampled schools that responded	92 (70.8)	94 (67.1)	48 (49.0)
Senior high	5115	4603	4907
Schools sampled	110 (2.2)	124 (2.7)	86 (1.8)
Sampled schools that responded	80 (72.7)	85 (68.5)	55 (64.0)
Total student responses	96 911	101 356	64 331
Excluded for nonresponse regarding sex or age	541	447	2
Excluded for discrepant responses (eg, sex, age, smoking, or drinking)	690	859	177
**Participant characteristics**	95 680	100 050	64 152
Sex			
Boys	48 525 (50.7)	51 587 (51.6)	34 582 (53.9)
Girls	47 155 (49.3)	48 463 (48.4)	29 570 (46.1)
School grade			
Junior high (aged 12-15 y)			
7	13 302 (13.9)	13 405 (13.4)	7384 (11.5)
8	13 649 (14.3)	12 884 (12.9)	7329 (11.4)
9	12 925 (13.5)	12 205 (12.2)	7415 (11.6)
Unknown	275 (0.3)	0	87 (0.1)
Senior high (aged 15-18 y)			
10	20 157 (21.1)	21 480 (21.5)	14 201 (22.1)
11	18 328 (19.2)	20 026 (20.0)	14 212 (22.2)
12	16 785 (17.5)	20 050 (20.0)	13 404 (20.9)
Unknown	259 (0.3)	0	120 (0.2)
Smoking and alcohol use in the past 30 d			
Smoking	4966 (5.2)	2851 (2.9)	1183 (1.8)
Alcohol use	16 110 (16.9)	12 034 (12.1)	3584 (5.6)

^a^ Unless indicated otherwise, values are presented as the No. (%) of schools or students.

Based on the 2008 surveys, 51.0% (95% CI, 50.9%-51.3%) of adolescents in grades 7 to 12 were exposed to SHS (≥1 day during the past 7 days) in any place; 37.2% (95% CI, 37.0%-37.5%) were exposed at home and 36.5% (95% CI, 36.3%-36.6%) were exposed in public places. In 2017, the prevalence decreased, but 36.3%, 23.8%, and 27.0% of participants were exposed to SHS in any place, at home, and in public places, respectively; 60.5% had spent a week in a smoke-free environment (eTable 1 in Supplement 2). The proportion of SHS exposure frequency in each survey is illustrated in eFigure 1 in Supplement 1. Regarding SHS exposure at home, the dominant exposure pattern was being exposed to SHS every day.

The association between SHS frequency and current smoking is presented in Table 2. The percentage of current smokers decreased from 2008 to 2017, but consistently increased with higher SHS exposure frequency. eTable 6 in Supplement 2 provides the missing data rates for each variable. The percentage of missing values across the 9 variables ranged from 0 to 5.7%. In total, there were 7676 instances (8.0%) of incomplete data in 2008, 3789 (3.8%) in 2012, and 3095 (4.8%) in 2017, for which we conducted multiple imputations. Exposure to SHS for 1 or more days during the past 7 days was associated with current smoking, regardless of survey year, sex, or location. The AORs for SHS exposure increased with higher exposure frequency (AORs ranged from 2.29 [95% CI, 1.81-2.91] for 1-2 days at home to 11.15 [95% CI, 8.50-14.62] for 7 days in public places). The AORs for SHS exposure increased with higher exposure frequency (Table 2). There was little difference in AORs across the survey years. Changes in these associations by sex were not distinct. The association between SHS exposure pattern and current smoking is shown in eTable 2 in Supplement 2. The AOR for current smoking compared with no exposure increased with the chance of exposure. These associations were consistent regardless of the sex or survey year.

**Table 2.** Association Between SHS Exposure Frequency and Current Smoking Among Adolescents in Grades 7 to 12 According to Exposure at Home and in Public Places^a^

Exposure to SHS within 7 d	2008			2012			2017		
	Current smokers	AOR (95% CI)	*P* value	Current smokers	AOR (95% CI)	*P* value	Current smokers	AOR (95% CI)	*P* value
Boys and girls									
At home, d									
0	1179/55 257 (2.1)	1 [Reference]	NA	812/68 471 (1.2)	1 [Reference]	NA	239/46 872 (0.5)	1 [Reference]	NA
1-2	405/6146 (6.6)	2.54 (2.23-2.89)	<.001	187/5120 (3.7)	2.65 (2.22-3.17)	<.001	61/2868 (2.1)	3.24 (2.36-4.45)	<.001
3-4	409/5021 (8.1)	3.08 (2.70-3.51)	<.001	305/5006 (6.1)	3.86 (3.31-4.51)	<.001	80/2669 (3.0)	4.39 (3.27-5.88)	<.001
5-6	356/4199 (8.5)	2.94 (2.55-3.39)	<.001	181/3078 (5.9)	3.42 (2.84-4.13)	<.001	50/1581 (3.2)	4.60 (3.22-6.57)	<.001
7	2349/20 262 (11.6)	3.67 (3.38-3.97)	<.001	1240/15 705 (7.9)	4.23 (3.82-4.69)	<.001	291/8143 (3.6)	4.21 (3.46-5.12)	<.001
In public places, d									
0	847/55 071 (1.5)	1 [Reference]	NA	593/68 098 (0.9)	1 [Reference]	NA	148/44 730 (0.3)	1 [Reference]	NA
1-2	748/15 157 (4.9)	2.69 (2.42-3.00)	<.001	363/12 344 (2.9)	3.07 (2.66-3.54)	<.001	108/7487 (1.4)	3.95 (3.03-5.17)	<.001
3-4	788/7906 (10.0)	4.80 (4.29-5.36)	<.001	602/7815 (7.7)	6.13 (5.39-6.98)	<.001	156/4646 (3.4)	6.88 (5.35-8.84)	<.001
5-6	599/3834 (15.6)	7.10 (6.26-8.05)	<.001	268/2612 (10.3)	7.66 (6.44-9.10)	<.001	71/1463 (4.9)	9.28 (6.69-12.87)	<.001
7	1712/8651 (19.8)	8.64 (7.85-9.52)	<.001	900/6544 (13.8)	9.46 (8.370-10.68)	<.001	239/3812 (6.3)	11.02 (8.73-13.90)	<.001
Boys									
At home, d									
0	861/28 240 (3.0)	1 [Reference]	NA	609/35 817 (1.7)	1 [Reference]	NA	179/25 396 (0.7)	1 [Reference]	NA
1-2	296/3078 (9.6)	2.64 (2.26-3.08)	<.001	138/2535 (5.4)	2.80 (2.27-3.45)	<.001	42/1478 (2.8)	3.26 (2.24-4.76)	<.001
3-4	276/2583 (10.7)	3.04 (2.59-3.58)	<.001	220/2534 (8.7)	3.92 (3.26-4.71)	<.001	65/1447 (4.5)	4.82 (3.46-6.70)	.01
5-6	237/2078 (11.4)	2.81 (2.36-3.34)	<.001	132/1540 (8.6)	3.63 (2.91-4.53)	<.001	37/774 (4.8)	4.98 (3.26-7.62)	<.001
7	1496/9661 (15.5)	3.54 (3.21-3.91)	<.001	824/7552 (10.9)	4.25 (3.76-4.80)	<.001	189/4050 (4.7)	4.11 (3.25-5.20)	.02
In public places, d									
0	616/29 059 (2.1)	1 [Reference]	NA	459/36 020 (1.3)	1 [Reference]	NA	114/24 708 (0.5)	1 [Reference]	NA
1-2	497/6690 (7.4)	2.84 (2.49-3.23)	<.001	253/5582 (4.5)	3.09 (2.61-3.65)	<.001	72/3472 (2.1)	3.92 (2.85-5.40)	<.001
3-4	484/3547 (13.6)	4.91 (4.27-5.64)	<.001	418/3753 (11.1)	6.32 (5.42-7.37)	<.001	112/2203 (5.1)	7.10 (5.28-9.54)	<.001
5-6	397/1809 (21.9)	7.67 (6.56-8.98)	<.001	177/1250 (14.2)	7.33 (5.93-9.07)	<.001	48/722 (6.6)	9.97 (6.73-14.77)	<.001
7	1175/4487 (26.2)	8.90 (7.92-10.00)	<.001	617/3431 (18.0)	9.28 (8.03-10.73)	<.001	167/2061 (8.1)	11.15 (8.50-14.62)	<.001
Girls									
At home, d									
0	318/27 017 (1.2)	1 [Reference]	NA	203/32 654 (0.6)	1 [Reference]	NA	60/21 476 (0.3)	1 [Reference]	NA
1-2	109/3068 (3.6)	2.29 (1.81-2.91)	<.001	49/2585 (1.9)	2.34 (1.67-3.28)	.02	19/1390 (1.4)	3.07 (1.73-5.47)	<.001
3-4	133/2438 (5.5)	3.15 (2.50-3.97)	<.001	85/2472 (3.4)	3.62 (2.73-4.81)	<.001	15/1222 (1.2)	2.86 (1.52-5.37)	<.001
5-6	119/2121 (5.6)	3.23 (2.54-4.09)	<.001	49/1538 (3.2)	2.88 (2.03-4.08)	.10	13/807 (1.6)	3.64 (1.85-7.18)	<.001
7	853/10 601 (8.0)	3.89 (3.37-4.48)	<.001	416/8154 (5.1)	4.04 (3.36-4.86)	<.001	102/4093 (2.5)	3.95 (2.77-5.64)	<.001
In public places, d									
0	231/26 012 (0.9)	1 [Reference]	NA	134/32 078 (0.4)	1 [Reference]	NA	34/20 022 (0.2)	1 [Reference]	NA
1-2	251/8467 (3.0)	2.39 (1.98-2.90)	<.001	110/6762 (1.6)	2.96 (2.26-3.86)	<.001	36/4015 (0.9)	3.81 (2.30-6.31)	<.001
3-4	304/4359 (7.0)	4.43 (3.67-5.34)	<.001	184/4062 (4.5)	5.73 (4.49-7.31)	<.001	44/2443 (1.8)	6.01 (3.67-9.82)	<.001
5-6	202/2025 (10.0)	6.07 (4.90-7.51)	<.001	91/1362 (6.7)	8.19 (6.04-11.09)	<.001	23/741 (3.1)	7.65 (4.20-13.93)	<.001
7	537/4164 (12.9)	7.94 (6.68-9.43)	<.001	283/3114 (9.1)	9.68 (7.69-12.20)	<.001	72/1751 (4.1)	9.19 (5.81-14.52)	<.001

Abbreviations: AOR, adjusted odds ratio; NA, not applicable; SHS, secondhand smoke.

^a^ Unless noted otherwise, data are presented as the No. of current smokers/No. of participants exposed to SHS (%). The following characteristics were adjusted in the analysis: sex and school grade, frequency of having breakfast, participating in club activities, enjoying school, college or higher education intention, understanding that secondhand smoking is harmful, understanding that smoking is harmful, and current alcohol drinking.

The association between SES and SHS exposure was also explored. The proportion of adolescents exposed to SHS was consistently lower in the group that intended to pursue college or higher education (eTable 3 in Supplement 2). The results of the stratified analysis according to 2 education intention levels are illustrated in the Figure and eFigure 2 in Supplement 1. The percentage of current smokers increased with the frequency of SHS exposure. Compared with the group with higher education intention, the percentage of current smokers was consistently higher in the group without higher education intention both at home and in public places. In addition, the increase in the percentage of current smokers with increasing SHS exposure frequency was more substantial in the group without higher education intention. From 2008 to 2017, the increase in the percentage of current smokers with increasing SHS exposure frequency flattened, and the difference between 0 days and 7 days decreased. Regardless of higher education intention, SHS exposure at home and in public places was associated with current smoking (eFigure 2 in Supplement 1). The association did not differ substantially between the 2 education intention levels. (Additional information regarding the association between selected covariates and current smoking or SHS exposure is provided in eTables 4-6 in Supplement 2.)

**Figure.**
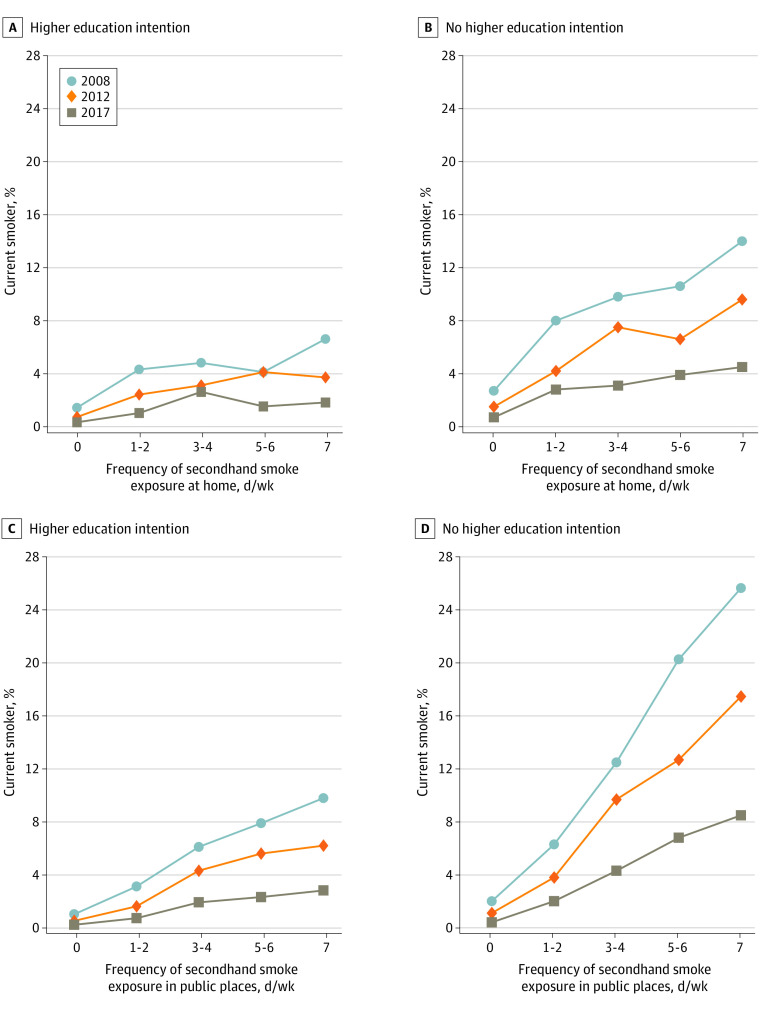
Prevalence of Current Smokers by Secondhand Smoke Exposure Frequency and Differences According to Higher Education Intention in 2008, 2012, and 2017

## Discussion

To our knowledge, this study is the first to assess SHS prevalence among adolescents in Japan. Our findings suggest that the percentage of adolescents exposed to SHS decreased between 2008 and 2017, which can be attributed to decreases in smoking rates, increases in smoking bans in workplaces and public places, increases in voluntary smoking restrictions at home and in workplaces, and changes in societal attitudes toward smoking around nonsmokers and children.^5^ However, in 2017, 1 in 3 adolescents (36.3%) in this study reported having been exposed to SHS for at least 1 day during the past 7 days, and 60.5% had spent a week in a smoke-free environment. Furthermore, about a quarter (23.8%) of junior high and high school students were exposed to SHS in their homes, and many were exposed to SHS almost every day. Although comparability was limited, the prevalence of SHS in our study was lower than the global figure from the Global Youth Tobacco Survey in 2010 to 2018 (62.9%)^11^ and about the same as that of nonsmoking children in 2013 to 2016 in a CDC report (35.4%).^13^ Our findings suggest that despite more than a decade of tobacco control efforts since FCTC ratification, SHS exposure remains a major public health issue. There is no safe threshold for SHS exposure; thus, stakeholders must ensure that all environments are free from unwanted SHS.^33^ Similar issues exist in Japan. To protect individuals from SHS, reducing the number of smokers by promoting effective and comprehensive tobacco control measures in accordance with the FCTC is a priority.

In this study, SHS exposure was consistently associated with smoking among adolescents themselves, regardless of location, frequency of exposure, or pattern of exposure. Many factors are interrelated with this association, including SHS exposure,^34,35^ parental modeling,^17,36^ and physical sensitivity to SHS.^37^ A meta-analysis reported that exposure to parental or sibling smoking is a notable risk factor for smoking uptake among children and adolescents, although this was not measured in our study.^38^ However, a finding from worldwide data shows an association between SHS and susceptibility to smoking.^39^ Secondhand smoke has been suggested to be an independent factor for susceptibility to smoking.^16^ Prospective studies have described SHS exposure at home as a more substantial risk factor than parental smoking,^17^ and it has also been reported to hamper smoking cessation.^40^ Although our cross-sectional study is insufficient to make causal inferences, SHS exposure may be an important factor in inducing youths to smoke. Moreover, in our study, SHS exposure in public places increased the magnitude of the association between SHS exposure and smoking. This result might be explained by reverse causality, as smokers tend to go to smoking areas. However, associated factors in the cross-sectional study can be detected as risk factors in the longitudinal study. Ensuring an environment without SHS has the potential to prevent youths from beginning to smoke and to reduce the number of future smokers.

In our stratified analysis by higher education intention, smoking rates among adolescents decreased from 2008 to 2017, regardless of higher education intention or the environment in which SHS exposure occurred. However, the prevalence of smoking and SHS exposure remained higher among adolescents who did not intend to pursue higher education. Smoking rates were lowest among adolescents free from SHS exposure, regardless of higher education intention. The changes observed suggest that SHS exposure decreased even among smokers over the study period. A decrease in parental smoking or local smoke-free legislation might have contributed to this finding. Additional findings suggest that the smoking environment, regardless of societal perspective, is closely linked to youth smoking. Moreover, the closer youths are to SHS in their environment, the greater their association with smoking. Protecting all children and adolescents from SHS through comprehensive smoke-free measures could contribute to a decrease in smoking rates regardless of social disparities. In the UK, children’s exposure to SHS decreased after legislation banned smoking inside public places.^41^ However, the effect of tobacco control interventions on socioeconomic disparities in smoking remains controversial.^42^ Previous studies have found a differential impact of smoke-free policies on different social groups.^43,44,45^ Evidence from Hong Kong showed that public smoke-free bans shifted smoking from public places to home, increasing home SHS exposure in children.^46^ Hence, prudent smoke-free regulations from multiple perspectives should be implemented to verify their effectiveness.

### Limitations

Several study limitations should be noted. First, our survey was based on self-reported responses; thus, it was difficult to examine their accuracy. However, both self-reported smoking behavior and SHS exposure among adolescents have adequate validity.^47,48,49^ Second, we included only adolescents who attended junior high and high schools in Japan on the day the survey was administered and who participated in the survey; therefore, participants were not representative of all individuals in this age group. However, in most countries, most young people in this age group attended school.^50^ Moreover, there is a possibility of bias associated with why some parents and caretakers may have chosen to not grant permission for their children’s participation in the study. However, it is unknown why some individuals did not respond and, notably, the number of nonresponses was minimal. Third, our estimates are conservative because we conducted a school-based survey, and students outside of school might be more likely to engage in risky behavior than those in school.^51^ Fourth, this cross-sectional study could not assess causality. Regarding the association between SHS and current smoking, we could not adjust for parental smoking because this information was not consistently collected for our sample. This could be an important source of unmeasured confounding factors. Fifth, our survey did not directly measure SES (eg, parental educational attainment or household income). Therefore, we used higher education intention as a marker for household SES, as suggested in previous literature. Sixth, the response rate changed, and biases may have been created. Although we could not evaluate the biases at this point, a way to improve the response rate should be explored in future studies.

## Conclusions

Our repeated cross-sectional study assessed trends in SHS exposure among Japanese adolescents from 2008 to 2017. These findings suggest that secondhand smoke exposure among adolescents has decreased in Japan; however, exposure to SHS among adolescents remains high. To decrease the number of smokers, enhancing comprehensive tobacco control strategies to meet the global standard is Japan’s first step in achieving a smoke-free environment. The association between SHS exposure frequency and current smoking was consistently observed regardless of survey year, location, or SES. Implementation and verification of the effectiveness of smoke-free legislation to reduce smoking initiation and health hazards for young people should be considered.

## Notes

Supplement 1. **eAppendix.** List of Questions From the Survey Questionnaire **eMethods.** Ethics Approval **eFigure 1.** Frequency of Secondhand Smoke Exposure at Home and in Public Places During the Past 7 Days by Sex and Age Group **eFigure 2.** Association Between Current Smoking and Secondhand Smoke Exposure Frequency and Its Difference According to Higher Education Intention in 2008, 2012, and 2017

Supplement 2. **eTable 1.** Prevalence of Secondhand Smoke Exposure in Any Place, at Home, and in Public Places Among Adolescents in Grades 7 to 12 According to Sex, Exposure Frequency, and Age Group **eTable 2.** Association Between Pattern of Secondhand Smoke Exposure and Current Smoking **eTable 3.** Prevalence of Secondhand Smoke Exposure at Home and in Public Places Among Adolescents in Grades 7 to 12 According to Higher Education Intention and Smoking Behavior **eTable 4.** Percentage of Current Smokers by Sex, School Grade, Having Breakfast, Participating Club Activity, Enjoying School, Higher Education Intention, Understanding Secondhand Smoking Is Harmful, Understanding Smoking Is Harmful, and Current Alcohol Use **eTable 5.** Percentage of Secondhand Smoking at Home by Sex, School Grade, Having Breakfast, Participating Club Activity, Enjoying School, Higher Education Intention, Understanding Secondhand Smoking Is Harmful, Understanding Smoking Is Harmful, and Current Alcohol Use **eTable 6.** Percentage of Secondhand Smoking in Public Places by Sex, School Grade, Having Breakfast, Participating Club Activity, Enjoying School, Higher Education Intention, Understanding Secondhand Smoking Is Harmful, Understanding Smoking Is Harmful, and Current Alcohol Use

Supplement 3.
Data Sharing Statement
