# Supplementary material for: Secondhand Smoke Exposure and Smoking Prevalence Among Adolescents
Source: JAMA Netw Open. 2023 Oct 20;6(10):e2338166. doi: 10.1001/jamanetworkopen.2023.38166 (PMC10589809; doi:10.1001/jamanetworkopen.2023.38166)
Supplement: Supplement 1. — eAppendix. List of Questions From the Survey Questionnaire eMethods. Ethics Approval eFigure 1. Frequency of Secondhand Smoke Exposure at Home and in Public Places During the Past 7 Days by Sex and Age Group eFigure 2. Association Between Current Smoking and Secondhand Smoke Exposure Frequency and Its Difference According to Higher Education Intention in 2008, 2012, and 2017 [file jamanetwopen-e2338166-s001.pdf]

# Supplementary Online Content

Kuwabara Y, Kinjo A, Kim H, et al. Secondhand smoke exposure and smoking prevalence among adolescents in Japan. *JAMA Netw Open*. 2023;6(10):e2338166. doi:10.1001/jamanetworkopen.2023.38166

**eAppendix.** List of Questions From the Survey Questionnaire

**eMethods.** Ethics Approval

**eFigure 1.** Frequency of Secondhand Smoke Exposure at Home and in Public Places During the Past 7 Days by Sex and Age Group

**eFigure 2.** Association Between Current Smoking and Secondhand Smoke Exposure Frequency and Its Difference According to Higher Education Intention in 2008, 2012, and 2017

This supplementary material has been provided by the authors to give readers additional information about their work.

© 2023 Kuwabara Y et al. *JAMA Network Open*.

**eAppendix.** List of Questions From the Survey Questionnaire

※**Students were asked to select one option for each question.**

1. **Demographic data**

- Are you male or female?

  - Male; Female

  (We used this binary variable as the binary data for adjustment).

- What is your grade in school?

  - Grade 1; Grade 2; Grade 3

  (We used this variable as discrete data for adjustment).

2. **Exposure to secondhand smoke**

- During the past 7 days, how many days have people smoked in your presence in your home?
  - 0 days; 1–2 days; 3–4 days; 5–6 days; 7 days
- During the past 7 days, how many days have people smoked in your presence, in places other than in your home?
  - 0 days; 1–2 days; 3–4 days; 5–6 days; 7 days

3. **Use of cigarettes**

- In the past 30 days, how many days have you smoked cigarettes?
  (In the 2017 survey, "cigarettes" was changed to "combustible cigarettes.")

  - 0 days; 1–2 days; 3–5 days; 6–9 days; 10–19 days; 20–29 days; Every day

- In the past 30 days, how many cigarettes per day have you smoked on average?

(In the 2017 survey, "cigarettes" was changed to "combustible cigarettes.")

  - Have not smoked in the past 30 days; Less than 1 cigarette per day; 1–4 cigarettes, 5–9 cigarettes; 10–14 cigarettes; 15–19 cigarettes; More than 20 cigarettes.

© 2023 Kuwabara Y et al. *JAMA Network Open*.

**4. Use of Alcohol**

- In the past 30 days, how many days have you consumed alcohol?

  - 0 days; 1–2 days; 3–5 days; 6–9 days; 10–19 days; 20–29 days; Every day

  (We categorized this into binary data for adjustment; Non-drinker: 0 days; or Current drinker: 1–2 days; 3–5 days; 6–9 days; 10–19 days; 20–29 days; Every day).

**5. Intention to pursue future education**

- What are your intentions after graduating from school? Choose the option closest to your current feelings.

  - Senior high school; Vocational school; 2-year-college; College; Graduate school; Start working; Not decided yet

  (We categorized this into binary data for adjustment; College or higher: College, Graduate school; or Other options; Senior high school, Vocational school, 2-year-college, Start working, Not decided yet).

**6. Attitude toward smoking**

1. Do you think that smoking is harmful to your body?

   - I do not think that smoking is harmful; I think that smoking might be a little harmful but not much; I think that smoking is harmful; I am not sure.

     (We categorized this into binary data for adjustment; Yes: I think that smoking might be a little harmful but not much, I think that smoking is harmful. No: I am not sure, I do not think that smoking is harmful).

2. Do you think that smoking from other people’s cigarettes is harmful to you?

   - I do not think it is harmful; I think it is probably not harmful; I think it is probably harmful; I think it is harmful.

   (We categorized this into binary data for adjustment; Yes: I think it probably is harmful, I think it is harmful. No: I do not think it is harmful, I think it is probably not harmful).

**7. Lifestyle behaviors and school life**

- Do you eat breakfast every day?

© 2023 Kuwabara Y et al. *JAMA Network Open*.

- I do almost every day; I do sometimes; I seldom do

(We categorized this into binary data for adjustment; Every day: I do almost every day. Not every day: I do sometimes, I seldom do).

- Do you participate in club activities?
    - I participate actively; I participate but not actively; I do not participate

    (We categorized this into binary data for adjustment; Yes: I participate actively, I participate but not actively. No: I do not participate).

- Do you enjoy school life?
    - Yes, it’s fun; I am not sure; No, it is not fun

    (We categorized this into binary data for adjustment; Yes: Yes, it’s fun. No: I am not sure, No, it is not fun).

© 2023 Kuwabara Y et al. *JAMA Network Open*.

**eMethods.** Ethics Approval

The participants were older than 12 years of age. Before the survey, the school principals provided participant parents with the survey details. The parents were advised that they could refuse participation if they were reluctant to allow their children to take part in the survey. In other words, the parents were given the opportunity to opt their children out of the survey, if they were not comfortable with their participation. The students whose parents refused permission for their participation in the survey were not included. This survey and opt-out parental consent procedure were approved by the Ethics Review Committee of Tottori University Faculty of Medicine (reference no. 17A078).

© 2023 Kuwabara Y et al. *JAMA Network Open*.

**eFigure 1.** Frequency of Secondhand Smoke Exposure at Home and in Public Places During the Past 7 Days by Sex and Age Group

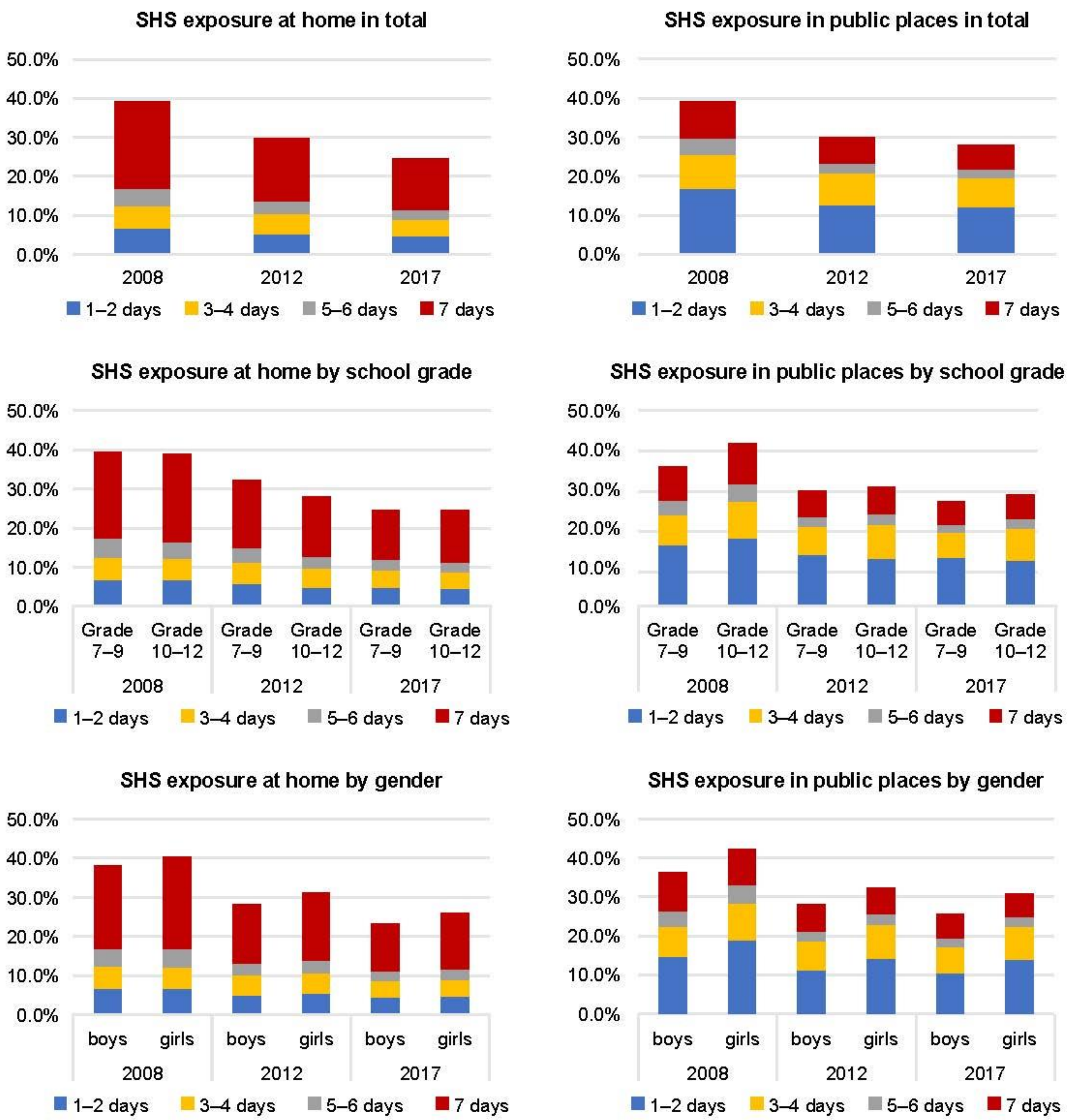


© 2023 Kuwabara Y et al. *JAMA Network Open*.

**eFigure 2.** Association Between Current Smoking and Secondhand Smoke Exposure Frequency and Its Difference According to Higher Education Intention in 2008, 2012, and 2017

**(The blue, orange, and gray lines represent adjusted odds ratio of current smokers by frequency of secondhand smoke exposure in 2008, 2012, and 2017).**

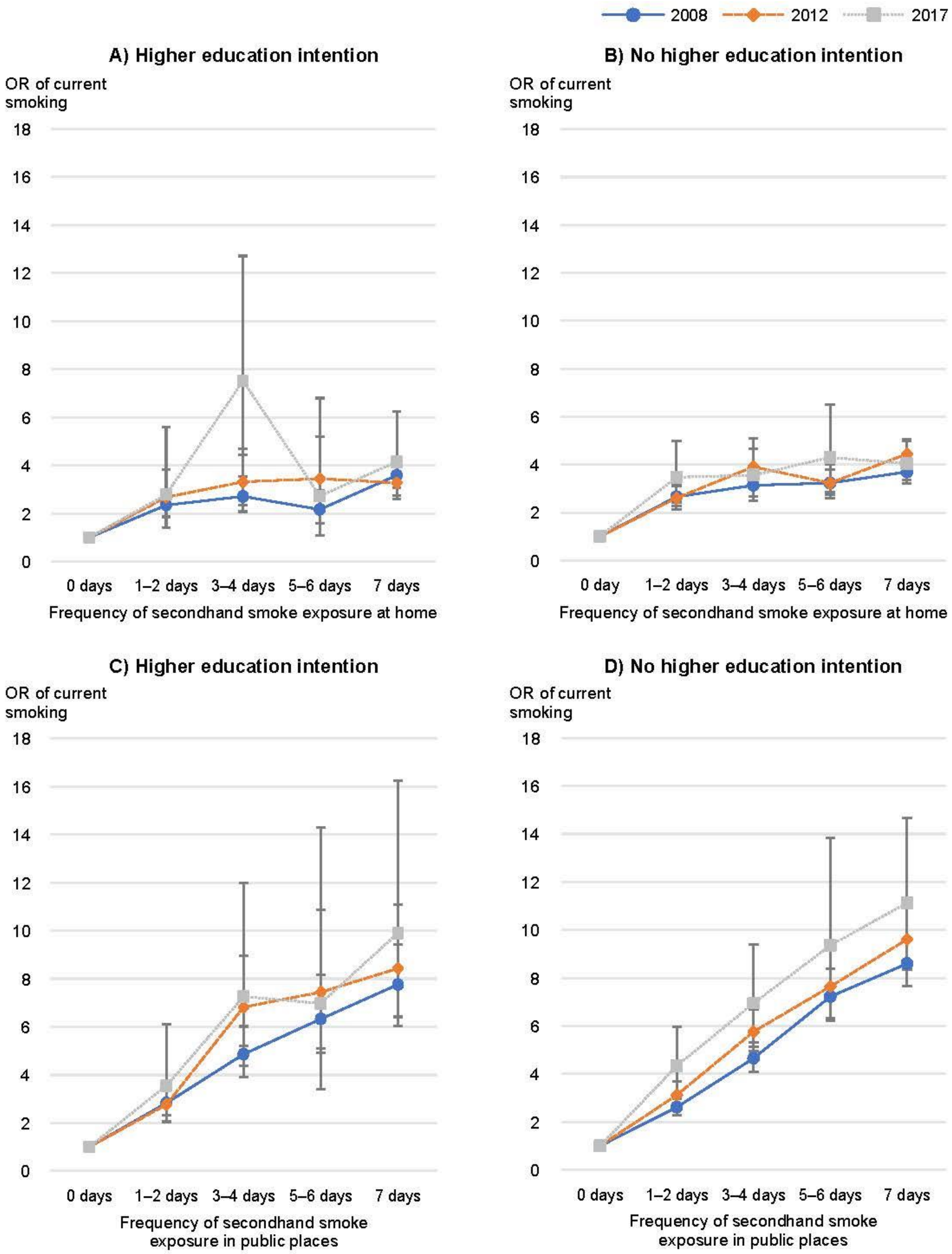


© 2023 Kuwabara Y et al. *JAMA Network Open*.
