# Supplementary material for: Secondhand Smoke Exposure and Smoking Prevalence Among Adolescents
Source: JAMA Netw Open. 2023 Oct 20;6(10):e2338166. doi: 10.1001/jamanetworkopen.2023.38166 (PMC10589809; doi:10.1001/jamanetworkopen.2023.38166)
Supplement: Supplement 3. — Data Sharing Statement [file jamanetwopen-e2338166-s003.pdf]

Data Sharing Statement

Kuwabara. Secondhand Smoke Exposure and Smoking Prevalence Among Adolescents in Japan. *JAMA Netw Open*. Published October 20, 2023.
doi:10.1001/jamanetworkopen.2023.38166

Data
Data available: No. The datasets generated and/or analyzed during this study are not publicly available because of the lack of consensus from other research members but are available from the corresponding author upon reasonable request.
